# RETRACTED: Li, S.; Chen, J. Synthesis and Properties of Novel Alkyl-Substituted Hexaazacyclophanes and Their Diradical Dications. *Molecules* 2024, *29*, 789

**DOI:** 10.3390/molecules30071545

**Published:** 2025-03-31

**Authors:** Shunjie Li, Jian Chen

**Affiliations:** 1 College of Chemistry and Materials Science, Anhui Normal University, Wuhu 241000, China; 2 College of Chemistry and Materials Science, Huaibei Normal University, Huaibei 235000, China

The *Molecules* Editorial Office retracts the article titled “Synthesis and Properties of Novel Alkyl-Substituted Hexaazacyclophanes and Their Diradical Dications” [1], cited above.

Following publication, the authors contacted the Editorial Office to raise concerns related to major scientific errors and an overlap between this publication [1] and a thesis [2] produced by a different author.

Adhering to our standard procedure, an investigation was conducted by the Editorial Office and Editorial Board, which confirmed the validity of concerns relating to the reproducibility of the central findings of this study and that data stemming from a thesis were published without appropriate permission or acknowledgment. As a result, the Editorial Board and authors have decided to retract this article [1] as per MDPI’s retraction policy (https://www.mdpi.com/ethics#_bookmark30).

This retraction was approved by the Editor-in-Chief of *Molecules*.

The authors agreed to this retraction.
